# Exosomes As Potential Biomarkers and Targeted Therapy in Colorectal Cancer: A Mini-Review

**DOI:** 10.3389/fphar.2017.00583

**Published:** 2017-08-28

**Authors:** Kha Wai Hon, Nadiah Abu, Nurul-Syakima Ab Mutalib, Rahman Jamal

**Affiliations:** UKM Medical Molecular Biology Institute, UKM Medical Centre, Universiti Kebangsaan Malaysia Kuala Lumpur, Malaysia

**Keywords:** exosomes, colorectal cancer, biomarkers, targeted therapy, molecular target

## Abstract

The number of colorectal cancer (CRC) cases have increased gradually year by year. In fact, CRC is one of the most widely diagnosed cancer in men and women today. This disease is usually diagnosed at a later stage of the development, and by then, the chance of survival has declined significantly. Even though substantial progress has been made in understanding the basic molecular mechanism of CRC, there is still a lack of understanding in using the available information for diagnosing CRC effectively. Liquid biopsies are minimally invasive and have become the epitome of a good screening source for stage-specific diagnosis, measuring drug response and severity of the disease. There are various circulating entities that can be found in biological fluids, and among them, exosomes, have been gaining considerable attention. Exosomes can be found in almost all biological fluids including serum, urine, saliva, and breast milk. Furthermore, exosomes carry valuable molecular information such as proteins and nucleic acids that directly reflects the source of the cells. Nevertheless, the inconsistent yield and isolation process and the difficulty in obtaining pure exosomes have become major obstacles that need to be addressed. The potential usage of exosomes as biomarkers have not been fully validated and explored yet. This review attempts to uncover the potential molecules that can be derived from CRC-exosomes as promising biomarkers or molecular targets for effective diagnosing of CRC.

## Introduction

Colorectal cancer is the fourth leading cause of cancer deaths globally with an estimation of 1.4 million new cases yearly around the world (Xu et al., 2016). Screening for the early detection of CRC is crucial to reduce its mortality through prevention and management before CRC progress into advanced stages (Us Preventive Services Task Force, 2016). Statistics have reported that the 5-year survival rate is up to 90% for CRC diagnosed at early stages and localized as compared to 71% for CRC cases with lymph node involvement, and only 13% when distant metastases are present (Howlader et al., 2016). Although there are multiple tests available for CRC screening, each method has its own limitations in terms of sensitivity and specificity. Currently, few available clinical biomarkers such as CEA and CA19-9 have been reported with low sensitivity and specificity for early detection of CRC (Nicolini et al., 2010; Lee et al., 2012; Su et al., 2012). Therefore, there is a demand for discovery of new biomarkers for better detection of CRC at early stages to improve the survival rate of patients. Throughout the years, many researchers have put much effort in CRC-related studies in order to understand the CRC pathogenesis, to explore potential markers which could be more sensitive for early diagnosis of CRC as well as to seek for new therapeutic options for CRC patients. Among all the new discoveries in CRC-related studies, EVs are starting to gain considerable interest from researchers around the globe.

Extracellular vesicles can be divided into three main categories based on the intracellular origins and/or biological functions, namely apoptotic bodies, microvesicles, and exosomes (El Andaloussi et al., 2013). In particular, exosomes are phospolipid bilayer nanovesicles that range from 50 to 100 nm in diameter (Wang et al., 2014). As compared to microvesicles (100 nm to 1 μm) and apoptotic bodies (1–5 μm), exosomes are the smallest EVs which are naturally secreted by almost every cell type including dendritic cells (Liu Q. et al., 2016), mast cells (Xiao et al., 2014), platelets (Goetzl et al., 2016), T lymphocytes (Wahlgren et al., 2012), epithelial cells (Borges et al., 2013), and neurons (Bahrini et al., 2015). Exosomes can be detected in all body fluids such as cerebrospinal fluid, plasma, urine, amniotic fluid, and even saliva (Keller et al., 2011; Lasser et al., 2011; Tietje et al., 2014). While microvesicles and apoptotic bodies form directly from outward budding of plasma membrane, exosomes are different to originate from MVBs within endocytic pathway (Bobrie et al., 2012). Among these three classes of EVs, exosomes are perhaps the best characterized subset of EVs. Pan and Johnstone (1983), exosomes were first reported to function as “garbage bags” secreted by sheep reticulocytes to remove obsolete materials. Since then, exosome biology has started to gain immense interest from the research community while numerous reports had revealed the functional importance of exosomes in various biological events, such as intracellular communication (Record et al., 2014), cell signaling (Xiao et al., 2014), tissue regeneration (Borges et al., 2013), immune response (Liu Q. et al., 2016), viral replication (Gallo et al., 2017), cancer development (Greening et al., 2015) as well as organ-specific metastasis (Hoshino et al., 2015) Evidently, exosomes have the unique ability of transporting different types of cargos including DNA, mRNA, miRNA, and proteins inside the same vesicles.

According to ExoCarta (as of March 2017) which is a database of exosomal proteins, RNAs and lipids, there are about 9700 proteins and 1110 lipids that are discovered to be associated with exosomes of various origins (Keerthikumar et al., 2016). The correlation between different components of exosomes and disease pathogenesis is still not fully elucidated as various findings from related studies have not been well-compared and coordinated toward a main concept. As compared to other classes of EVs, exosomes as intracellular messengers have relatively high abundancy and stability in circulating entities to carry genetic information and other biological materials, which could be useful as biomarkers and therapeutic targets (Yoon et al., 2014; Milane et al., 2015; Zhang et al., 2015). At present, there is still no exosome-based clinical testing approved for diagnostic, prognostic and predictive purposes of CRC cases. Therefore, this review will specifically discuss the potential roles of exosomes as biomarkers in CRC as well as to provide insight into the future direction for exosome-related research.

## Composition of Exosomal Membrane

The proteomic and biochemical analysis of purified exosomes have also revealed that the phospolipid bilayer membrane of exosomes is embedded with various proteins and lipids which originate from the parent cells. These exosomal proteins and lipids may serve as surface markers for the characterization and differentiation of exosomes from other types of microvesicles besides having potential roles in various biological interactions. Generally, endosomal proteins such as Alix, TSG101, calthrin, and ubiqutin are highly conserved among exosomes from most of the cell types (Bobrie et al., 2012). These proteins are components of the ESCRT-I complex, which assist in the sorting of endocytic ubiquitinated cargos into MVBs (early precursors of exosomes) besides acting as mediators of the association between ESCRT-0 and ESCRT-I complex (Baietti et al., 2012; Sun et al., 2015; Christ et al., 2016). Exosomes are also enriched with integrins and tetraspanins that are transmembrane proteins responsible for cellular targeting and adhesion, also commonly used as molecular markers to distinguish from other classes of microvesicles (Rana et al., 2012). Integrins on tumor exosomes may play important roles in modulating organ-specific metastasis as keystone of cancer progression (Hoshino et al., 2015). CD9, CD63, and CD81 are few tetraspanins commonly detected on all types of exosomes (Wang et al., 2014). CRC-exosomes have been reported to carry tetraspanin CD24 which is an important biomarker commonly detected in many maglinancies including ovarian cancer (Im et al., 2014) and CRC (Nosrati et al., 2016). Coincidentally, exosomes extracted from blood plasma of CRC patients showed upregulation in CD24 expression, suggesting its possible implication in early diagnosis of CRC (Yunusova et al., 2016). CD147 is another novel tetraspanin that has been detected on exosomes released by CRC cell lines as well as in patient serum by using Exoscreen method, which could be a new rapid and highly sensitive detection method (Yoshioka et al., 2014).

Heat shock proteins namely HSC70, HSP60, HSP70, and HSP90 frequently found in all exosomes are proposed to modulate protein trafficking into ILVs which are precursors of exosomes (Lv et al., 2012). Annexins and Rab proteins selectively found in certain subsets of exosomes are important for exosome formation, membrane trafficking and release of exosomes from parent cells (Kastelowitz and Yin, 2014). Certain cytoskeleton proteins can be detected in exosomes, including actin, tubulin, and cofilin (Kowal et al., 2014). Additionally, exosomes are commonly enriched in lipid-rafts including cholesterol, sphingolipids, ceramide, and glycerophospholipids containing long and saturated fatty-acyl chains (Record et al., 2014). As exosomes are mostly embedded with host cell-specific transmembrane membrane, exosomes derived from the human colon carcinoma cell line LIM1215 have been shown to carry colon epithelial cell-specific A33 antigen and EpCAM as distinctive cellular markers (Tauro et al., 2013). The A33 protein is a glycoprotein that is highly expressed in CRC and been used in clinical trials for targeted therapy (Welt et al., 2003; Baptistella et al., 2016). The expression of A33 protein is also positively correlated with the differentiation status of CRC (Baptistella et al., 2016). EpCAM or CD326, has been shown to be highly overexpressed in most of the CRC tissues, and significantly correlated with abnormal cell proliferation, invasion, and metastases of CRC (Liu et al., 2014). High level of EpCAM expression on LIM1215-derived exosomes could be optimized as a specific antigen in developing antibody microarray for detection of cancer cell lines based on surface proteins of exosomes (Belov et al., 2016). On the other hand, higher level of circulating exosomes correlated with poor prognosis and shorter survival time were observed in CRC patients as compared to healthy controls, which could be used as tumor indicator for CRC cases but further investigation is required to study the release mechanism of exosomes into the plasma of CRC patients (Silva et al., 2012).

## Intracellular Proteins in CRC-Exosomes

Only a handful of studies have been performed to analyze the total protein content of CRC-exosomes. An interesting study by Mathivanan et al. (2010) revealed that both A33-exosomes and EpCAM-exosomes carry some other tissue-specific proteins such as cadherin-17, CEA, PCNA, EGFR, mucin 13, misshapen-like kinase 1, keratin 18, mitogen-activated protein kinase 4, claudins (1, 3, and 7), centrosomal protein 55 kDa, and ephrin-B1 and -B2. For the first time, it has been proposed that cadherin-17 ectodomain in LM1215-derived exosomes is more specific than CEA in early detection of CRC (Bernhard et al., 2013). Cadherin-17 is a calcium-dependent transmembrane glycoprotein that has been reported to be overexpressed in gastric, pancreatic, and colorectal adenocarcinomas (Su et al., 2008). Meanwhile, exosomes derived from CRC cell lines were found to be enriched with TGF- β, which is essential to inhibit immune response against CRC cells by suppressing T cell proliferation and transform phenotype of T cells into tumor-growth supporting cells (Yamada et al., 2016)

Chen et al. (2017) have performed quantitative proteomics analysis of SPEs from 36 patients with primary CRC (stages I, II, and III) and 49 healthy volunteers, in which they managed to discover a total of 58 proteins with 36 of them upregulated while another 22 were downregulated. Their findings suggested that those upregulated proteins take part in ECM remodeling, cell communication, and signal transduction as well as the enhancement of vascular permeability and tumor-promoting inflammation. A few examples of upregulated proteins found in SPEs from CRC patients are SERPINA1, SERPINF2, and MMP9 (Chen et al., 2017). In contrast, those downregulated proteins found are related to immune escape of tumor growth, complement binding, complement activity, growth factor activity and cellular adhesion, implicating that CRC-derived, SPEs may exert minimal alteration in tumor survival and proliferation. Examples of downregulated proteins include ILK, CAPNS1, and NRAS (Chen et al., 2017). Most of these proteins are known to be upregulated in cancer tissues but in CRC-exosomes they were down-regulated. This implicates that the protein components of the exosomes do not always reflect the parent cells.

**FIGURE 1:**
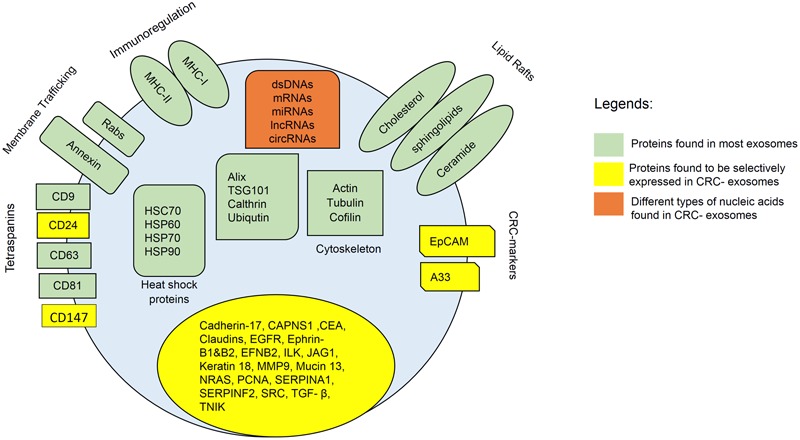
Composition of CRC-exosomes. All exosomes carry multiple types of proteins, including major histocompatibility complex (MHC)-II, cluster of differentiation (CD), tetraspanins, heat shock protein (Hsp), Ras-related protein (Rab), signal transduction proteins, and etc. Exosomes also contain different types of lipids such as cholesterol, ceramide, and etc. In addition, exosomes contain various types of nucleic acids, including dsDNA, miRNA, mRNA, and non-coding RNAs.

Notably, it has been demonstrated that exosomes derived from metastatic SW620 human colon cancer cells display a proteomic profile significantly different from non-metastatic primary CRC cell exosomes (Ji et al., 2013). Metastatic CRC cell exosomes are particularly enriched in metastatic factors, signal transduction molecules, lipid raft and lipid raft-associated components, providing valuable information on the metastatic keystone for CRC patients. However, more extensive studies are required to discover and understand about the total intracellular proteins in exosomes secreted by different types of parent cells. **Figure 1** summarizes the proteins that can be found in general exosomes and CRC-derived exosomes.

## MicroRNAs in CRC-Exosomes

Tumor cells also secrete exosomes to transport multiple genetic materials and biological cargos which are essential to maintain the tumoral microenvironment and influence cancer progression. Tumor-derived exosomes can even travel to distant sites to induce metastatic niches and immune suppression which further favors the aggressive transformation of cancerous cells (Wang et al., 2014). MicroRNAs are small non-coding RNAs that specifically inhibit mRNA translation or induce mRNA degradation, which could be essential in the initiation and development of cancers when tumor suppressor genes are suppressed. Exosomal microRNAs have been targeted as potential biomarkers for the diagnosis of CRC at initial stages due to the wide availability and high specificity of certain exosomal microRNAs to CRC (Ogata-Kawata et al., 2014). Serum level of exosomal miR-17-92a and miR-19a have been found to be upregulated in CRC patients including the recurrent cases (Matsumura et al., 2015). Overexpression of miR-17-92a as an oncogenic miRNA in CRC patients is highly correlated with tumorigenesis especially at the early stages of cancer development (Concepcion et al., 2012). Elevated level of miR-19a has been reported to promote cancer cell proliferation and tumor invasion at any stage of CRC and is possibly correlated with poor prognosis (Zheng et al., 2014). Meanwhile, a recent study has reported that exosomes released from human colon cancer cells carry miR-210 that may influence the adhesion of neighboring metastatic cells (Bigagli et al., 2016). Originally, miR-210 is upregulated in CRC tissues while its overexpression is strongly correlated with aggressive invasion and distant metastasis (Qu et al., 2014). Increased levels of exosomal miR-193a were observed in CRC patients especially those with liver metastasis in advanced stages, implicating the possible role of miR-193a to promote tumor progression (Teng et al., 2017)

Other microRNAs in CRC-exosomes such as let-7a, miR-1229, miR-1246, miR-150, miR-223, and miR-23a have been reported with significant increment as compared to exosomes from healthy subjects (Ogata-Kawata et al., 2014). It has been proposed that exosomal microRNAs can achieve high specificity and sensitivity of up to 95% for miR-1229 while the sensitivities of CA19-9 and CEA for stage I CRC are only 10 and 15% respectively, suggesting the potential of exosomal microRNAs as novel biomarkers in diagnosing CRC even at early stages. Another study had utilized next generation sequencing to characterize exosomal microRNAs in two major classes of exosomes (A33 and EpCAM-positive respectively) that were derived from human LIM1863 colon cancer cell line (Ji et al., 2014). The results reported two different miRNA expression profiles when certain microRNAs can only be detected in exosomes from specific cell lines. Such implications may attract more attention for more related work to be carried out in the future as it shows that even within the same CRC pathogenesis, the molecular profile within the exosomes remain different. Although there are various microRNAs being discovered in CRC-exosomes, currently there is still no collective view of which microRNAs should be selected for CRC.

## Other Classes of Nucleic Acids in CRC-Exosomes

mRNAs and lncRNAs are long RNAs with the length of 200 nucleotides and above. Emerging evidence has proposed that lncRNAs are crucial regulators in various biological processes involved in carcinogenesis (Han et al., 2015). Among all three classes of EVs, exosomes are the most enriched with lncRNAs (Dong et al., 2016). In addition to this, few exosomal lncRNAs namely CRNDE-h and MAGEA3 have been identified as potential biomarkers for CRC detection (Dong et al., 2016; Liu T. et al., 2016). For instance, exosomal CRNDE-h was found to be positively correlated with poor prognosis of CRC patients, suggesting the high value of exosomal CRNDE-h as a key prognostic factor in CRC cases (Liu T. et al., 2016).

Circular RNAs (circRNAs) are a set of non-coding RNAs that are covalently closed from the 5′ end to the 3′ end (Abu and Jamal, 2016). CircRNAs are abundant, differentially expressed in different diseases, more stable than linear RNAs and have longer half-lives (Abu and Jamal, 2016). Recently, circRNAs are found to be more enriched in exosomes than in parent cells (Li et al., 2015; Dou et al., 2016; Lasda and Parker, 2016). The presence of circRNAs in CRC-exosomes has been reported in CRC cell lines and serum of CRC patients (Li et al., 2015; Dou et al., 2016). Additionally, the enrichment of circRNAs in KRAS mutant cells is significantly downregulated as compared to KRAS wild-type cells. Among the differentially expressed exosomal circRNAs found in KRAS mutant CRC cell lines were circFAT1, circARHGAP5, and circHIPK3 (Dou et al., 2016). On the other hand, in serum-derived exosomes from CRC patients, the enrichment of circRNAs differ between CRC patients and healthy subjects. In CRC patients, some of the circRNAs were missing and new circRNAs were identified (Li et al., 2015). Li et al. (2015) discovered that circ-KLDHC10 was significantly upregulated in CRC-derived exosomes as compared to exosomes from healthy controls. Since circRNAs are generally more enriched and stable in exosomes, circRNAs may serve as promising biomarkers for CRC cases (Li et al., 2015).

Additionally, ΔNp73 mRNA is reported to be enriched in CRC-exosomes and can be transferred into a recipient cell (Soldevilla et al., 2014). Upon reception, the CRC-derived exosomes containing ΔNp73 mRNA may affect the acceptor’s cell proliferation and drug resistance response. This indicates that the components within an exosomal boundaries not only contain passive information but may also actively regulate recipient cells directly. Exosomes derived from human SW480 CRC cells were reported with enrichment in 27 cell cycle-related mRNAs which may promote proliferation of endothelial cells to induce angiogenesis in tumor growth and metastasis (Hong et al., 2009). Interestingly, HCT-116 CRC cells-derived exosomes were shown to contain internal double stranded DNA (Thakur et al., 2014). Exosomal DNA may serve as a translation biomarker for early detection of cancer especially in regard to the parental cell mutation status (Thakur et al., 2014). **Table 1** lists down all of the nucleic acids and proteins found in CRC-derived exosomes.

**Table 1:** Summary of studies that have investigated differentially expressed exosomal nucleic acids in CRC.

Class	Name	Sources	Expression	Potential role	Reference
miRNAs	miR-17-92a	Serum	Upregulated	Early stage tumorigenesis	Matsumura et al., 2015
	miR-19a	Serum		Promote cancer cell proliferation and tumor invasion	
	let-7a	Serum	Upregulated	Not specific	Ogata-Kawata et al., 2014
	miR-1229				
	miR-1246				
	miR-150				
	miR-223				
	miR-23a				
	miR-193a	Plasma		Promote tumor progression	Teng et al., 2017
	miR-210	Cell line		Distant metastasis	Bigagli et al., 2016
mRNAs	ΔNp73	Serum	Upregulated	∙ Affect proliferation and drug resistance ∙ Related to advanced stage of CRC and shorter disease-free survival	Soldevilla et al., 2014
lncRNAs	CRNDE-h	Serum	Upregulated	Poor prognosis of CRC	Liu T. et al., 2016
	MAGEA3			Not specific	Dong et al., 2016
circRNAs	circ-KLDHC10	Cell line and serum	Upregulated	Not specific	Li et al., 2015
	circRTN4	Cell line			Dou et al., 2016
	CircFAT1				
	circARHGAP5				
	CircHIPK3				
Proteins	CD 24	Plasma	Upregulated	Early detection of CRC	Yunusova et al., 2016
	A33	Cell line	Enriched	Differentiation of CRC	Tauro et al., 2013
	CD 147			Early detection of CRC	Yoshioka et al., 2014
	EpCAM				Tauro et al., 2013
	Cadherin-17				Bernhard et al., 2013
	CEA			Clinical biomarker of CRC	Mathivanan et al., 2010
	PCNA			Not specific	
	EGFR				
	TGF-β			Immune escape	Yamada et al., 2016
	SERPINA1 SERPINF2 MMP9	Serum	Upregulated	ECM remodeling, cellular communication, signal transduction, vascular permeability, inflammation	Chen et al., 2017

## Bottlenecks, Future Direction, and Conclusion

The study of exosomes in the pathogenesis of CRC has started to gain considerable attention from the research community. Most of the current studies mainly focus on developing exosomal biomarkers for early detection and prognosis prediction of CRC. However, as mentioned earlier, the lack of standardization and optimisation for most of the current exosomes purification protocols remains as one of the major challenges for CRC-related exosomal studies. Also, the physical features as to what constitutes an “exosome” are diverse and are still, by standard, elusive. This is a major challenge if exosomes are to be used as biomarkers, for clinical diagnosis of different diseases. As researchers begin to understand more about the potential roles of CRC-exosomes, the future direction of related studies may lie within a broader scope of research such as the recruitment or activation of the immune system to counteract CRC tumorigenesis, tailored drug delivery systems, biomarkers for metastasis prediction and even targeted elimination of exosomes-mediated metastasis. Based on our review of many studies so far, even within an exosomal vesicle, there are various molecules that can be used as biomarkers or potential targets for therapy. Proteins and nucleic acids found in CRC-exosomes can serve as biomarkers for CRC whether in combination with each other or individually. However, as mentioned above, even within the same pathogenesis, the molecular profile of the exosomes may be different. For future screening or profiling studies, exosomes from the same source can be first divided based on their extracellular protein profile and only then be separated based on the components within. Nevertheless, a more efficient way of classifying exosomes should be developed either by the protein profile, nucleic acid profile, size or affinity to better utilize exosomes as biomarkers or targeted therapy. Therefore, more effort is required to translate the diverse properties of exosomes into the development of highly sensitive diagnostic strategies for rapid and non-invasive diagnosis as well as better prognosis prediction of CRC cases.

## Author Contributions

KWH and NA: drafted and wrote the manuscript, NA: conceived the idea for the manuscript, NA, NSAM, and RJ: provided critical analysis and language editing.

## Conflict of Interest Statement

The authors declare that the research was conducted in the absence of any commercial or financial relationships that could be construed as a potential conflict of interest. The reviewer DL and handling Editor declared their shared affiliation.

## Abbreviations

Alix: ALG-2-interacting protein X
CA19-9: carbohydrate antigen 19-9
CAPNS1: calpain small subunit 1
CEA: carcinoembryonic antigen
CircRNAs: circular RNAs
CRC: colorectal cancer
ECM: extracellular matrix
EGFR: epidermal growth factor receptor
EpCAM: epithelial cell surface antigen
ESCRT: endosomal sorting complex required for transport
EVs: extracellular vesicles
ILK: integrin-linked protein kinase
ILVs: intraluminal vesicles
LncRNAs: long non-coding RNAs
MMP9: matrix metalloproteinase-9
MVBs: multivesicular bodies
NRAS: GTPase NRas
PCNA: proliferating cell nuclear antigen
SERPINA1: alpha-1-antitrypsin
SERPINF2: alpha-2-antiplasmin
SPEs: serum-purified exosomes
TGF- β: transforming growth factor-β1
TSG101: tumor susceptibility gene 101
